# Study of Photochemical Cytosine to Uracil Transition via Ultrafast Photo-Cross-Linking Using Vinylcarbazole Derivatives in Duplex DNA

**DOI:** 10.3390/molecules23040828

**Published:** 2018-04-04

**Authors:** Siddhant Sethi, Shigetaka Nakamura, Kenzo Fujimoto

**Affiliations:** Department of Advanced Science and Technology, Japan Advanced Institute of Science and Technology, 1-1 Asahidai, Nomi, Ishikawa 923-1211, Japan; siddhant@jaist.ac.jp (S.S.); s-nakamu@jaist.ac.jp (S.N.)

**Keywords:** deamination, C to U transition, cyanovinylcarbazole, photo-cross-linking, genome editing

## Abstract

Gene therapies, including genome editing, RNAi, anti-sense technology and chemical DNA editing are becoming major methods for the treatment of genetic disorders. Techniques like CRISPR-Cas9, zinc finger nuclease (ZFN) and transcription activator-like effector-based nuclease (TALEN) are a few such enzymatic techniques. Most enzymatic genome editing techniques have their disadvantages. Thus, non-enzymatic and non-invasive technologies for nucleic acid editing has been reported in this study which might possess some advantages over the older methods of DNA manipulation. 3-cyanovinyl carbazole (^CNV^K) based nucleic acid editing takes advantage of photo-cross-linking between a target pyrimidine and the ^CNV^K to afford deamination of cytosine and convert it to uracil. This method previously required the use of high temperatures but, in this study, it has been optimized to take place at physiological conditions. Different counter bases (inosine, guanine and cytosine) complementary to the target cytosine were used, along with derivatives of ^CNV^K (^NH2V^K and ^OHV^K) to afford the deamination at physiological conditions.

## 1. Introduction

Nucleic acid chemistry and its biological applications have great potential for developing futuristic drugs and curing many diseases [1]. Enzymatic methods for nucleic acid manipulation, such as PCR-plasmid based DNA manipulation, have been used for many years to create directed mutations, recombination, deletion and insertion of desired genes in the genome but these methods have limited applications in vivo [2]. Recent developments in the field of genome editing have given us methods for genome editing using proteins, RNA and other chemical agents. Currently, the major enzymatic techniques used for genome editing are meganuclease [3], zinc finger nuclease (ZFN) [4], transcription activator-like effector-based nuclease (TALEN) [5] and clustered regularly interspaced short palindromic repeats-associated system (CRISPR-Cas system) [6,7,8,9].

Currently, the CRISPR-Cas system is considered one of the most advanced techniques for specific genetic manipulation [10]. CRISPR-Cas9 is a two-component based system, wherein one of the components is an endonuclease (Cas9) and a guide RNA (gRNA), a 20-nucleotide sequence that determines target specificity [11]. Despite the high specificity of the CRISPR system, unintended mutations at sites other than target sites are one of the major drawbacks of the system [12].

Along with CRISPR, ZFN and TALEN have been used for genome editing by using enzymes that cleave the DNA at specific sites to induce double stranded breaks, which lead to deletion and frame-shift mutations [13,14]. Although useful, these systems have shown cytotoxicity and the cost of producing the enzymes is high [15]. Besides enzymatic methods for DNA manipulation, chemical methods such as sodium bisulphite nucleobase editing have been developed [16]. Bisulphite based nucleobase editing is disadvantageous as it requires deleterious chemicals and the technique is highly non-specific. Due to limitations in the chemical methods, certain photochemical methods were developed that use azobenzene and psoralen incorporated in the DNA strand and could undergo either photoisomerization or photo-cross-linking by UV irradiation to form a stable cross-linked duplex [17,18,19,20]. These photoactive compounds have been used in the DNA to induce anti-sense effects and form stable DNA structures [21,22,23].

Consecutively, new non-enzymatic and non-invasive techniques for manipulating nucleic acids have been reported. In 2010, we reported on this enzyme-free technique for nucleic acid editing that takes advantage of cytosine deamination to induce single point mutations in the DNA/RNA sequence using photoreactive 3-cyanovinylcarbazole nucleotides (^CNV^K) [24,25]. Upon incorporation of ^CNV^K into oligodeoxyribonucleic acid (ODN), the ODN becomes responsive towards UV radiation and can undergo cross-linking with cytosine at −1 position with respect to ^CNV^K in a sequence specific manner. Upon cross-linking, the aromaticity of cytosine is lost, making it prone to nucleophilic attack by water to undergo deamination. The major drawback of this reaction is the requirement of high temperature to accomplish deamination.

In 2015, we showed the effect of hydrogen bonding on the rate of the photo-cross-linking reaction by changing the counter-base of cytosine [26], which led to the idea that hydrogen bonding could also play a role in the deamination reaction. In 2017, we further proved our hypothesis by showing that certain counter-bases, especially inosine, of cytosine are better for deamination than other bases due to a specific type of hydrogen bonding in the cross-linked cytosine [27]. Furthermore, we also reported that not only hydrogen bonding, but also the polarity around the cytosine-photo-adduct, is responsible for the velocity of the deamination reaction at lower temperatures using derivatives of vinylcarbazole [28].

**Figure 1:**
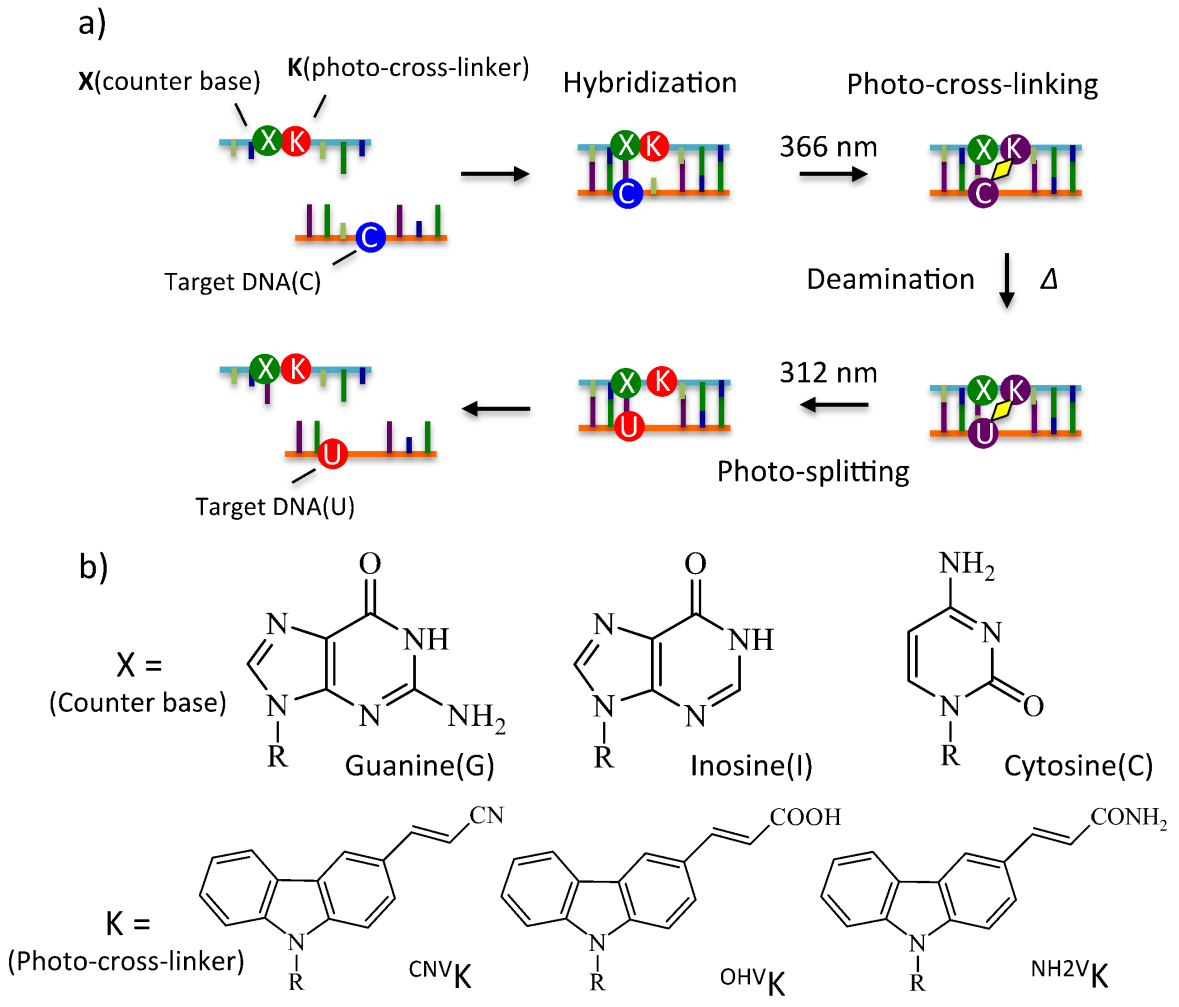
(**a**) Overview of photo-cross-link based cytosine deamination. (**b**) Chemical structures of counter bases and photo-cross-linkers.

In this report, we have shown the combined effect of hydrogen bonding and polarity by taking varying combinations of counter-bases (inosine, guanine and cytosine) and vinylcarbazole derivatives (3-cyanovinyl carbazole, 3-amidovinylcarbazole and 3-carboxyl vinyl carbazole) and incorporating them in the ODN to accomplish deamination at physiological conditions (Figure 1).

## 2. Results and Discussion

In order to find the best combination of photo-active nucleobase and counter base of the target, various combinations were studied for deamination. For the counter base: inosine, guanine and cytosine were chosen. For the photo-active nucleobase, 3-cyanovinyl carbazole (^CNV^K), along with two derivatives of ^CNV^K: 3-amidevinyl carbazole and 3-carboxyl vinyl carbazole were chosen. In our previous reports, these bases showed promising results when studied individually, therefore, we chose the combinations based on the previous singular studies. Firstly, 15mer ODNs containing various combinations (Table 1), namely: ODN(G^CNV^K), ODN(G^NH2V^K), ODN(G^OHV^K), ODN(C^CNV^K), ODN(C^NH2V^K), ODN(C^OHV^K), ODN(I^CNV^K), ODN(I^NH2V^K) and ODN(I^OHV^K), were synthesized using a DNA synthesizer (AB 3600, Foster City, CA, USA). The ODNs were designed so the counter base gets placed complementary to the target cytosine and the photo-active nucleotide gets placed at the −1 position with respect to the target cytosine present in the complementary strand.

**Figure 2:**
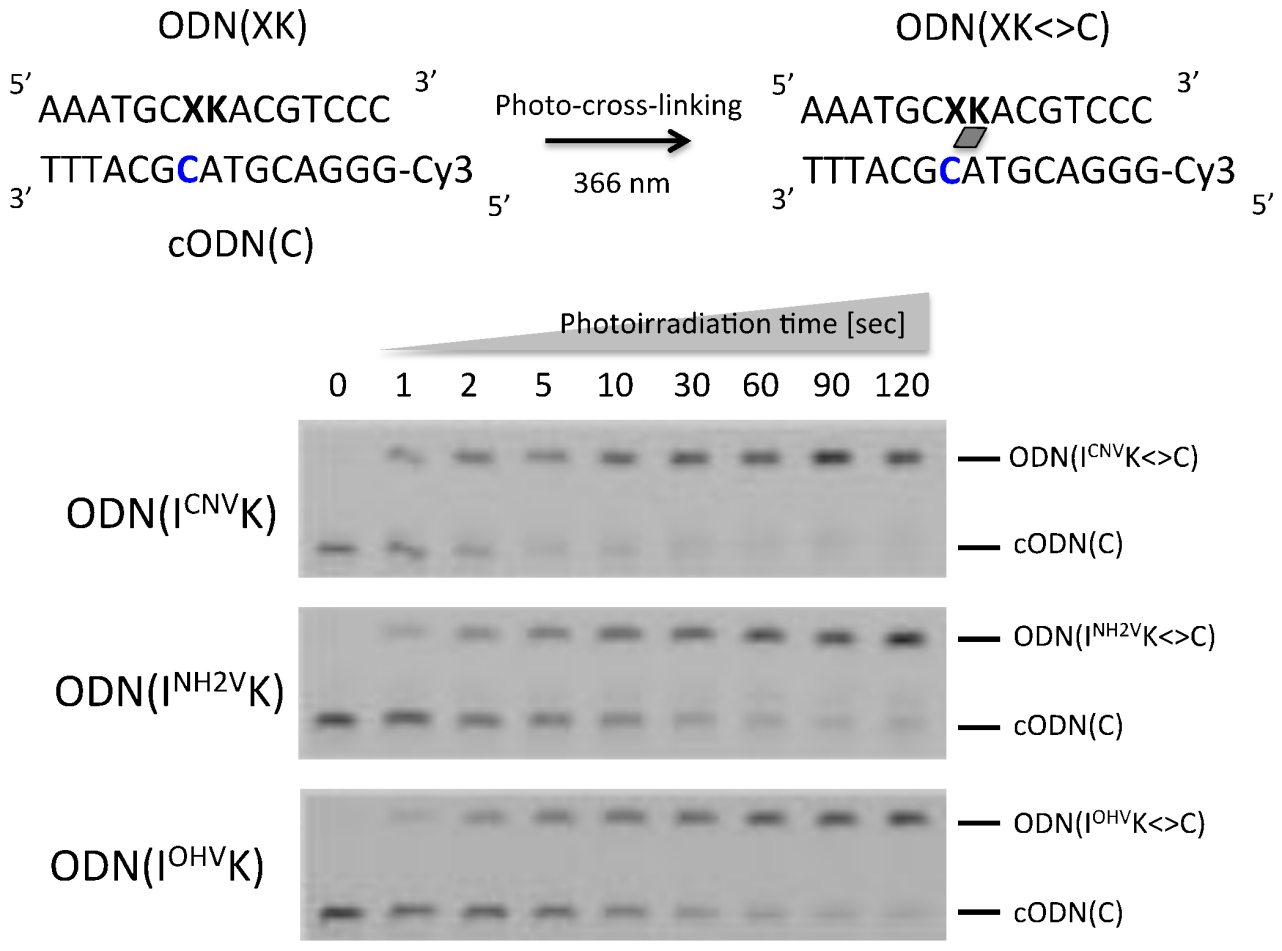
Polyacrylamide gel electrophoresis (PAGE) analysis of the mixture of ODN(XK) and Cy3-modified cODN(C) after photo-cross-linking using various times. [ODN] = 10 µM in 50 mM sodium cacodylate buffer (pH 7.4) containing 100 mM NaCl. Photoirradiation at 366 nm was performed at 4 °C. Ultra-high performance liquid chromatography (UPLC) chromatograms for photo-cross-linking are shown in Figure S1.

The two ODNs, Cy3-modified cODN(C) and ODN(XK) (10 µM each), where X = G, C and I; and K = ^CNV^K, ^NH2V^K and ^OHV^K, in 50 mM sodium cacodylate buffer containing 100 mM sodium chloride (pH 7.4) were irradiated with 366 nm UV radiation for 120 s at 4 °C using UV-LED(ZUV-C10, Kyoto, Japan). The solutions were analyzed by 15% denaturing Polyacrylamide gel electrophoresis (PAGE) and the result is shown in Figure 2. In case of ODN(I^CNV^K), ODN(I^NH2V^K) and ODN(I^OHV^K), The band corresponding to cODN(C) decreased depending on photoirradiation time and the band of ODN(XK<>C) appeared. Then all ODNs were cross-linked in 60 s with a yield of >90% as analyzed by denaturing PAGE analysis. These photoadducts were purified using high performance liquid chromatography (HPLC).

**Figure 3:**
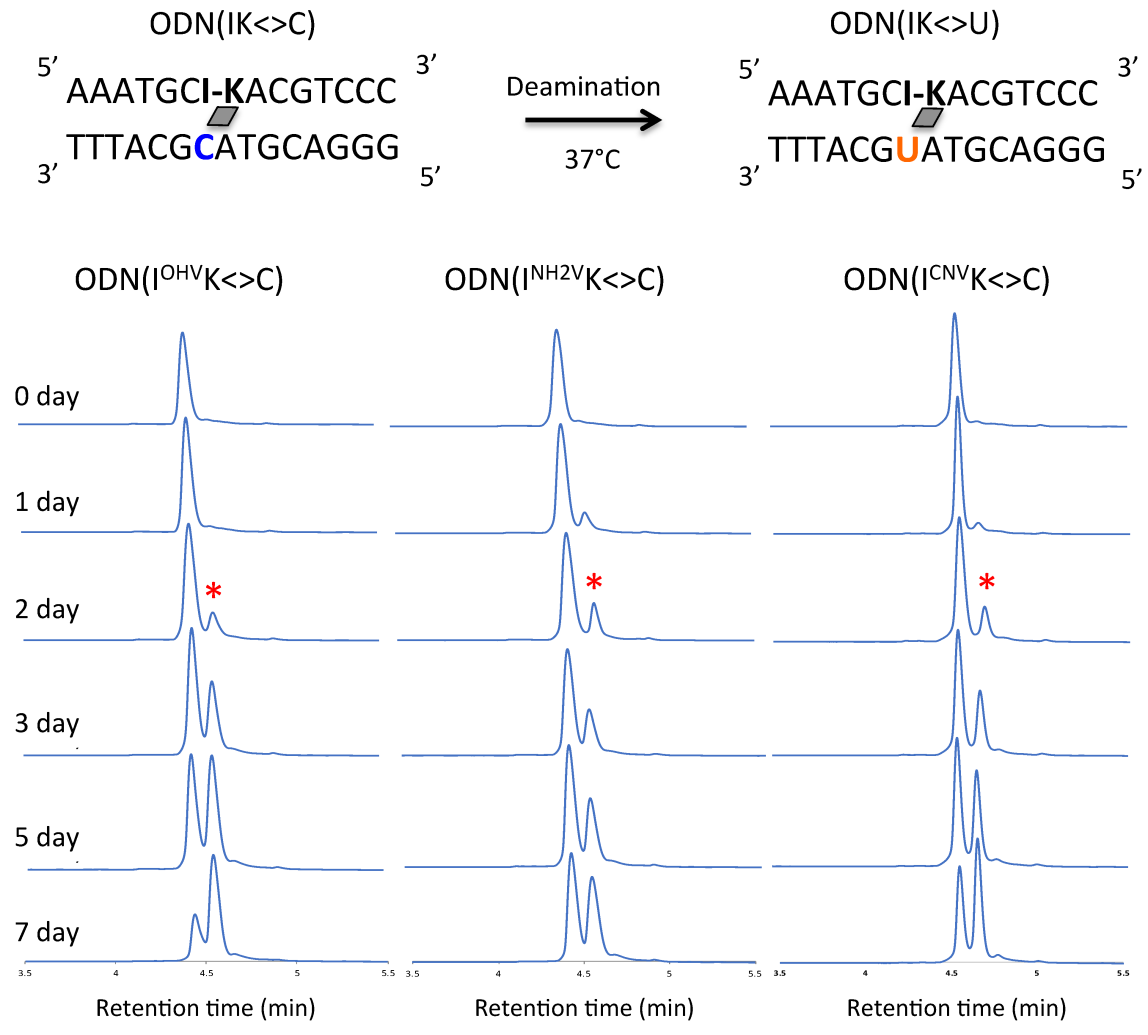
UPLC analysis of the deamination reaction of the photo-cross-linked duplex consisting of ODN(IK) and cODN(C). [ODN(IK<>C)] = 5 μM in 50 mM Na-cacodylate buffer (pH 7.4) containing 100 mM NaCl. incubated at 37 °C, photo splitting was performed with transilluminator (312 nm) at 37 °C. Peak marked with asterisk (*) indicate the newly formed ODN(U).

**Figure 4:**
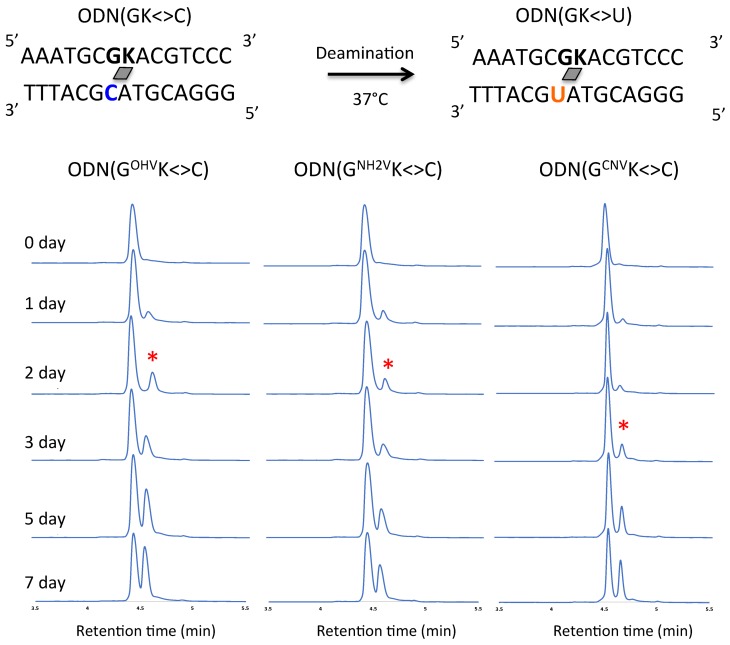
UPLC analysis of the deamination reaction of the photo-cross-linked duplex consisting of ODN(GK) and cODN(C). [ODN(GK<>C)] = 5 μM in 50 mM Na-cacodylate buffer (pH 7.4) containing 100 mM NaCl. incubated at 37 °C, photo splitting was performed with transilluminator (312 nm) at 37 °C. Peak marked with asterisk (*) indicate the newly formed ODN(U).

**Figure 5:**
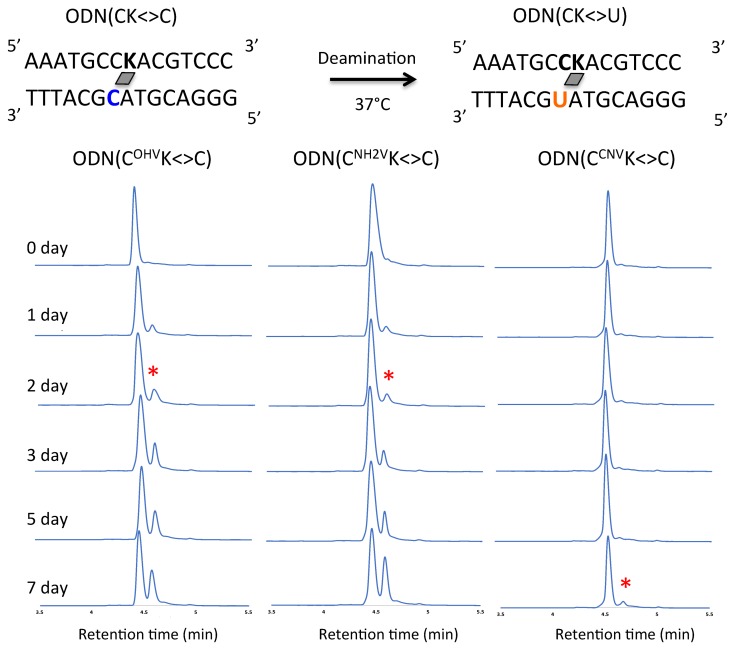
UPLC analysis of the deamination reaction of the photo-cross-linked duplex consisting of ODN(CK) and cODN(C). [ODN(CK<>C)] = 5 μM in 50 mM Na-cacodylate buffer (pH 7.4) containing 100 mM NaCl. incubated at 37 °C, photo splitting was performed with transilluminator (312 nm) at 37 °C. Peak marked with asterisk (*) indicate the newly formed ODN(U) with asterisk indicate the newly formed ODN(U).

The ODN(XK<>C) (5 µM) in 50 mM sodium cacodylate buffer and 100 mM sodium chloride (pH 7.4) were incubated for 7 days at 37 °C. Samples were removed at 1 day intervals and the ODNs were photo-split using 312 nm UV radiation for 15 min at 37 °C. The photo-split ODNs were then analyzed by Ultra-high performance liquid chromatography (UPLC). The conversion of C→U was studied by comparing peak areas under the curves of UPLC depicting the individual cODN(C) and cODN(U) shown in the Figure 3, Figure 4 and Figure 5. The photo-splitted ODNs were also subjected to enzymatic digestion using Nuclease P1 and alkaline phosphatase to digest the ODNs to a single nucleotide and to confirm the deamination reaction (Figure S2). In the case of inosine as a counter base, deamination occurred by incubation at 37 °C using ODN(I^OHV^K), ODN(I^NH2V^K) and ODN(I^CNV^K). The peak identical to ODN(C) was observed before incubation. The peak decreased upon incubation and a new peak identical to ODN(U) appeared (Figure 3). Surprisingly, in the case of ODN(I^OHV^K<>C), deamination occurred approximately 70% upon incubation at 37 °C for a week. We observed that deamination proceeded at the maximum rate (*k* = 7.1 × 10^−3^ h^−1^) in the case of ODN(I^OHV^K<>C). Deamination reaction rates were intermediate when ODN(I^NH2V^K<>C) and ODN(I^CNV^K<>C) were used, with *k* = 3.5 × 10^−3^ h^−1^ and 4.7 × 10^−3^ h^−1^, respectively. It is interesting to note here that the rate of deamination while using ODN(I^CNV^K) in 7 days is 4.7 × 10^−3^ h^−1^, whereas, while using ODN(I^OHV^K) the rate has increased almost 1.6 folds. This has definitely shown an improvement over our previous results where we obtained ~35% conversion in 3 days [27]. Also, with ODN(I^OHV^K<>C), the rate of deamination of cytosine to uracil was 2-fold faster than that with ODN(I^NH2V^K<>C). In the case of guanine as a counter base, the deamination occurred by incubation at 37 °C using ODN(G^OHV^K<>C), ODN(G^NH2V^K<>C) and ODN(G^CNV^K<>C). The peak identical to ODN(C) was observed before incubation. The peak decreased upon incubation and a new peak identical to cODN(U) appeared (Figure 4).

In the case of cytosine as counter base, deamination occurred by incubation at 37 °C using ODN(C^CNV^K<>C), ODN(C^OHV^K<>C) and ODN(C^NH2V^K<>C). A peak identical to that of ODN(C) was observed before incubation. A peak decreased on incubation and a new peak identical to ODN(U) appeared (Figure 5). It can be noted that the highest conversion of C→U was achieved by ODN(I^OHV^K<>C) (~70%) in 7 days. In general, among all the counter-bases, inosine containing ODNs showed maximum C→U conversion in combination with the photo-cross-linkers. ODNs containing cytosine and guanine showed almost similar reaction rates for all the photo-cross-linkers. The difference in the rate of deamination in the photo-cross-linked ODNs can be attributed to hydrogen bonding pattern. Due to cross-linking, the overall planarity of cytosine is perturbed and a tilt appears in the non-planar part of cytosine towards the central axis pertaining to change in the hydrogen bonding pattern. These changes lead to a hydrogen bond between the adjacent base of cytosine and the amino group of cytosine, specifically observed in case of ODN with inosine. These hydrogen bonding patterns are comparable to hydrogen bonding observed near the active site of yeast cytosine deaminase (yCD), wherein a special network of hydrogen bonds has been observed in the enzyme [29,30]. This special network of hydrogen bonding is critical for the protonation of N3 of cytosine through the Glu64 and conformational changes of cytosine giving accessibility to water attack facilitated by zinc-bound water on C4 of cytosine. The patterns observed in the cross-linked cytosine of hydrogen bonding are equivalent to these patterns observed in yCD. Prior to cross-linking, the hydrogen bonding is according to Watson-Crick base-pairing, whereas, after cross-linking, due to change in planarity of cytosine, the hydrogen is with adjacent base facilitating the nucleophile attack on C4. Furthermore, the hydrogen bonding N3 is also crucial for proton shuttling.

**Figure 6:**
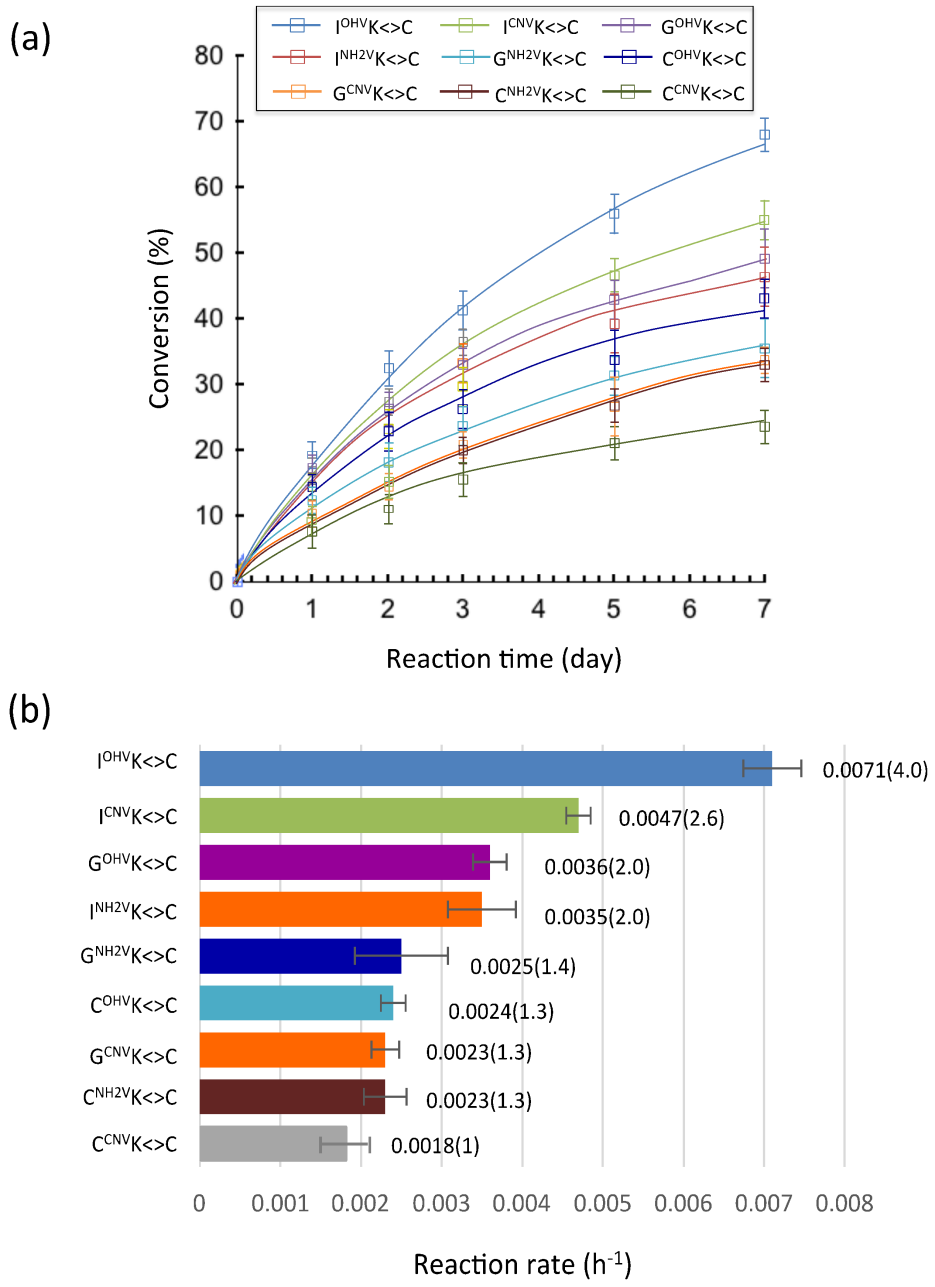
(**a**) Time course of the deamination reactions of the photo-adducts ODN(XK<>C). (**b**) Deamination reaction rate constants of the photo-adducts ODN(XK<>C). The values besides the bars are reaction rate constants and the values in braces indicate the acceleration ratio compared to the case of C^CNV^K. Photoreaction rate constants were estimated from the time course of the deamination reaction with an assumption of first-order reaction kinetics.

**Figure 7:**
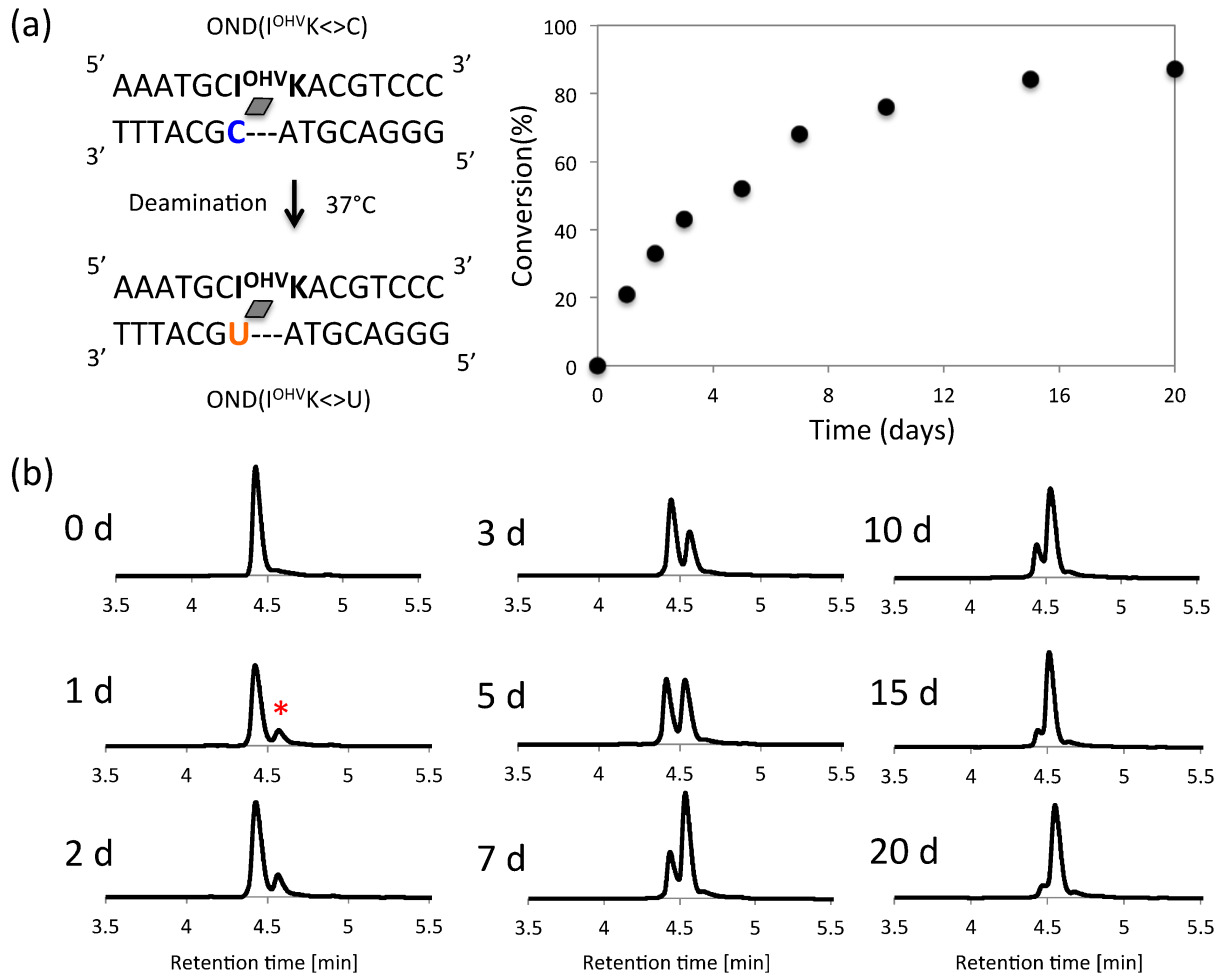
(**a**) Scheme and sequence of deamination and time course of deamination reaction. (**b**) UPLC analysis of the deamination reaction of the photo-cross-linked duplex consisting of ODN(IK) and cODN(C) for a duration of 20 days. [ODN(IK<>C)] = 5 μM in 50 mM Na-cacodylate buffer (pH 7.4) containing 100 mM NaCl. incubated at 37 °C, photo splitting was performed with transilluminator (312 nm) at 37 °C. Peak marked with asterisk (*) indicate the deaminated product.

Therefore, the number of hydrogen bonds in the photoadduct directly affected the rate of deamination, wherein fewer H-bonds between the target cytosine and counter-base facilitated the deamination reaction. Upon comparing the photo-cross-linkers, the same pattern was observed. In this photochemical deamination, the hydrophilicity around the C4 in the target cytosine is the important factor because of cytosine deamination requires water molecules. To assess the correlation between the hydrophilicity of photo-cross-linked dsDNA and rate of deamination reaction, we calculated the partition coefficient (LogP) [31,32] of photo-cross-linked dsDNA using their HPLC retention times (Table 2). The hydrophilicity of ODNs positively correlated with their ability to convert cytosine to uracil in a photo-responsive manner. Photo-cross-linked ODN(I^OHV^K<>C), ODN(G^OHV^K<>C) and ODN(C^OHV^K<>C) have the highest hydrophilicity value (LogP = 0.52, 0.54 and 0.57 respectively) among all the ODNs due to presence of highly hydrophilic photo-cross-linker ^OHV^K (Table 2 and Figure S3). Among these ODNs, it has been observed that the highest rate of cytosine to uracil conversion has been observed in ODN(I^OHV^K<>C) having the highest hydrophilicity. Similar pattern for deamination of cytosine has been observed in the ODNs containing ^NH2V^K. The highest rate has been observed in ODN(I^NH2V^K<>C) followed by ODN(G^NH2V^K<>C) and ODN(C^NH2V^K<>C) (Figure 6), having their LogP in the similar order, that is, highest hydrophilicity is shown by ODN(I^NH2V^K<>C) among these three. Even in the ODNs with ^CNV^K, the relation between LogP (hydrophilicity) and deamination reaction rate constant is positive correlation (Table S2 and Figure S4). ODNs containing ^OHV^K are the most hydrophilic therefore shows highest rate of cytosine to uracil conversion. Thus, upon careful analysis of the results it is evident that the two major factors required for high rate of deamination in the vinyl carbazole based photo-cross-link assisted cytosine deamination are hydrogen bonding in the target cytosine and the hydrophilicity of the photo-cross-linker. Upon combining these two factors, the maximum C→U conversion can be achieved by using ODN containing inosine and ^OHV^K at physiological conditions, therefore ODN(I^OHV^K<>C) having the highest hydrophilicity and optimum hydrogen bonding has shown highest rate of deamination among all the other ODNs. Thus, ODN(I^OHV^K<>C) was then further incubated for 20 days at 37 °C to determine the extent of deamination that can be achieved in this period. It has been observed from the UPLC chromatogram that the deamination reached a stationary phase and the reaction proceeded only minimally in the later stage of deamination (Figure 7).

In this paper, 7 days of reaction at physiological conditions gives ~70% conversion, which is optimum for in vivo applications under physiological conditions. These results give hope that, in the future, this non-enzymatic technique of site-directed mutagenesis might be used in vivo for curing and treating various disorders arising because of mutations in the genome.

## 3. Materials and Methods 

### 3.1. General

1H nuclear magnetic resonance (NMR) spectra were recorded on a AVANCE III 400 system (Bruker, Billerica, MA, USA). Mass spectra were recorded on a Voyager PRO-SF, (Applied Biosystems, Foster City, CA, USA). HPLC was performed on a Chemcosorb 5-ODS-H column with JASCO PU-980, HG-980-31, DG-980-50 system equipped with a JASCO UV 970 detector (JASCO, Tokyo, Japan) at 260 nm. Reagents for DNA synthesis such as A, G, C, T-β-cyanoethyl phosphoramidite and CPG support were purchased form Glen Research (Sterling, VA, USA).

### 3.2. Synthesis and Preparation of Modified Oligonucleotides

**Scheme 1:**
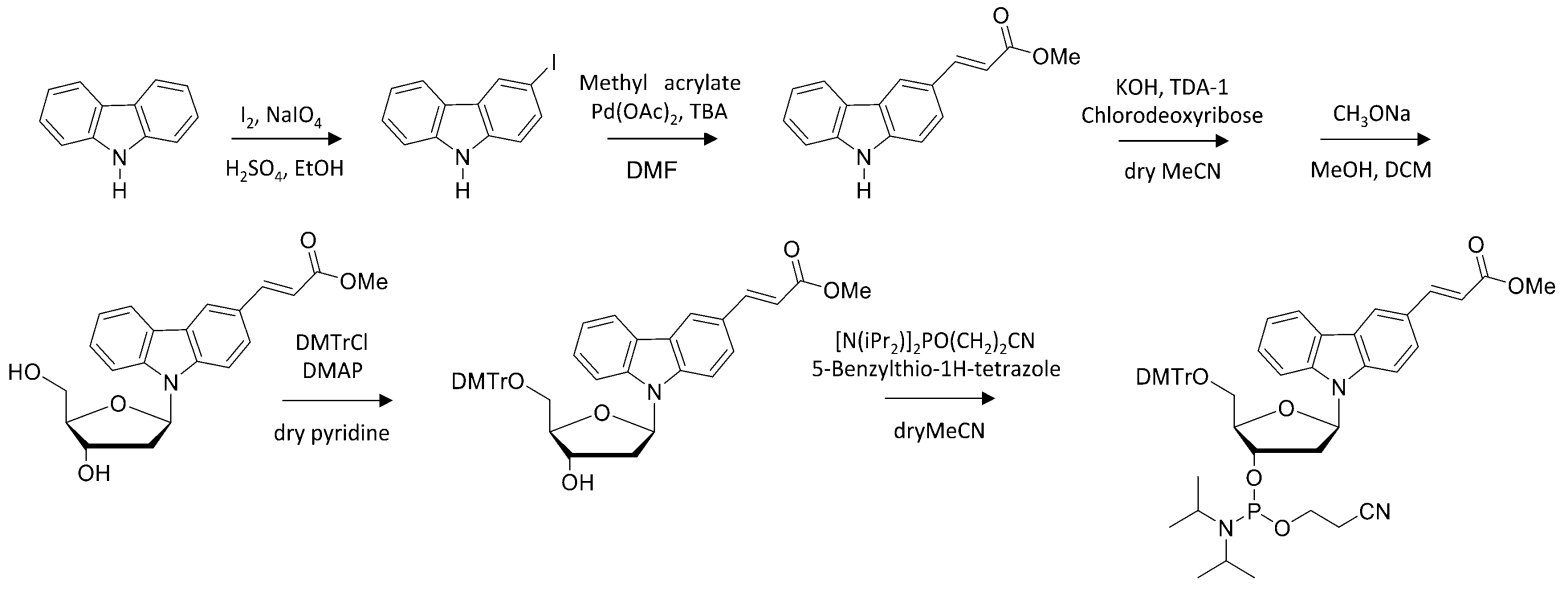
Synthetic scheme for preparation of photo-responsive nucleotide.

**Scheme 2:**
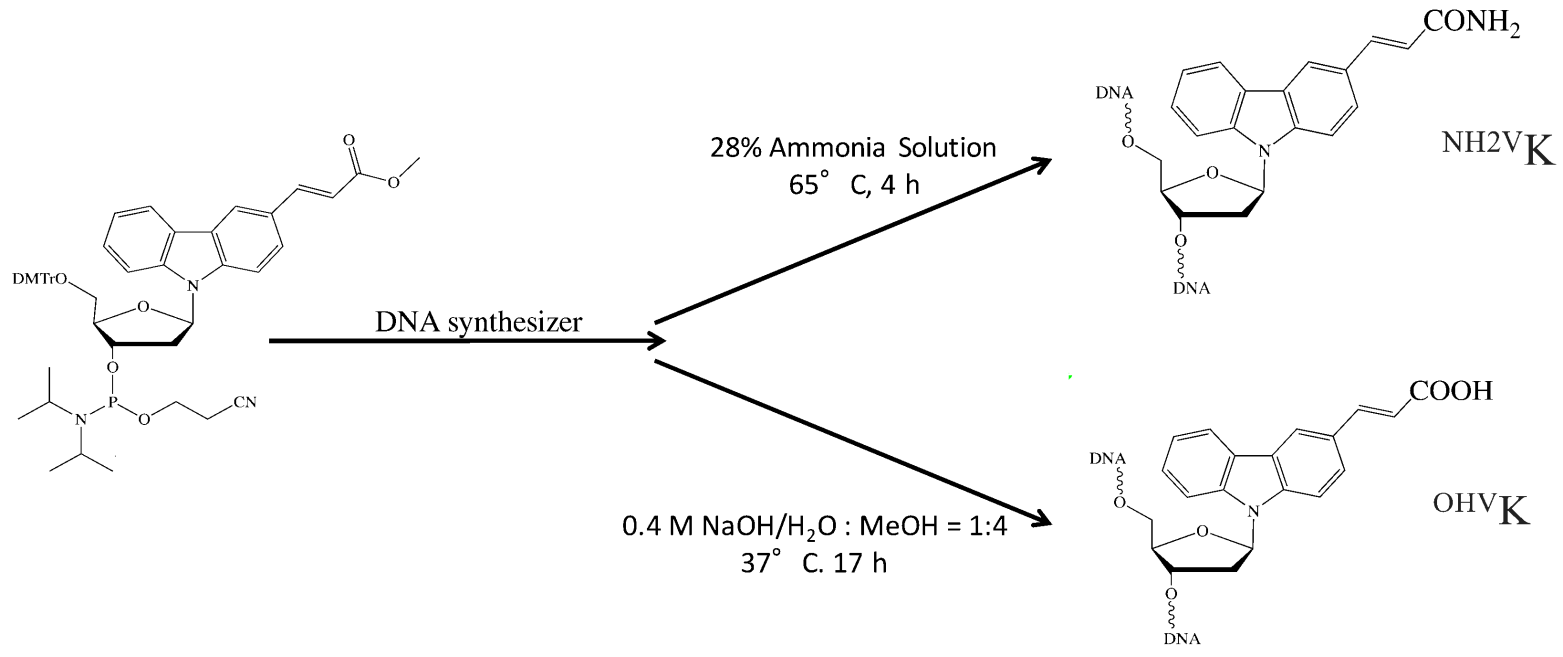
Post-modification of photo-cross-linker in ODN.

The phosphoramidite of ^OMeV^K and ^CNV^K were prepared following to Scheme 1 and previous reports [28,33]. The modified oligonucleotides containing ^CNV^K or ^OMeV^K were prepared, per standard phosphoramidite chemistry using DNA synthesizer (ABI 3400 DNA synthesizer, Applied Biosystems, Foster City, CA, USA). The ODN containing ^OMeV^K was post modified to ODN containing ^OMeV^K, ^OHV^K, or ^NH2V^K in deprotection step (Scheme 2). Synthesized ODN were detached from the support by soaking in concentrated aqueous ammonia for 1 h at room temperature. Deprotection was conducted by heating the aqueous concentration for 4 h at 65 °C, before removing the concentrated aqueous ammonia by speedvac and purifying the crude oligomer by reverse phase HPLC equipped with InertSustainTM C18 column Cosmosil^TM^ 5C_18_-AR-II column (5 μm, 10 × 150 mm, (Nacalai tesque, Kyoto, Japan), Flow rate of 3.0 mL/min, 60 °C) and lyophilizing it. Synthesis of ODN was confirmed by MALDI-TOF-MS. Other ODNs were purchased from Fasmac (Kanagawa, Japan).

### 3.3. Denaturing PAGE Analysis

Polyacrylamide gel electrophoresis (PAGE) was performed with 15% polyacrylamide containing 8M urea. After the electrophotresis (150 V, 80 min), a fluorescent image was taken by luminescent image analyzer (LAS3000, Fujifilm, Tokyo, Japan).

### 3.4. Isolation of Photo-Cross-Linked dsDNA

The cODN (C) (15 μM) and ODN (XK), where X is the counter base and K is the photo-cross-linker, (15 μM) in a buffer solution (100 mM NaCl, 50 mM sodium cacodylate, pH 7.6), was photoirradiated at 366 nm for 120 s using UV-LED illuminator (OMRON Inc., Tokyo, Japan, 1600 mW) at 37 °C. And the solution was purified by reverse phase HPLC and the concentration of photo-cross-linked dsDNA was determined by absorbance at 260 nm.

### 3.5. Deamination

The solution of 15 μM ODN(XK) and ODN(C) in buffer solution was annealed and photoirradiated at 366 nm. The photoirradated solution was purified by a reversible HPLC to obtain the purified photo-cross-linked dsDNA. The 5 μM photo-cross-linked dsODN in a buffer solution (50 mM Na-cacodylate buffer (pH 7.4) containing 100 mM NaCl) was incubated at 37 °C.

### 3.6. Photo-Splitting and UPLC Analysis

After the photo-splitting with irradiation of 312 nm (15 min at 37 °C, transillluminator, (Funakoshi, Tokyo, Japan)), the reaction mixture was analyzed with the UPLC system (Aquity, Waters, Milord, MA, USA); elution was with 0.05 M ammonium formate containing 3–6.5% CH_3_CN, linear gradient (10 min) at a flow rate of 0.2 mL/min.

### 3.7. Partition Coefficient (LogP)

LogP of photo-cross-linked DNA was measurement by their retention time following OECD protocol. 10 μM photo-cross-linked dsDNA was analyzed with HPLC system; elution was with 0.05 M ammonium formate containing 98–50% CH_3_CN, linear gradient (60 min) at flow rate of 1 mL/min. 4-Acetylpyridine, Aniline, Acetanilide, Phenol, Benzonitrile and Acetophenone were used as control compound to create calibration curve [26,27].

### 3.8. Enzymatic Digestion

ODNs after photo-splitting were incubated with 0.2 unit/µL Nuclease P1 for 24 h at 37 °C. Further, the solution was treated with alkaline phosphatase (0.1 unit/µL) for 24 h at 37 °C and analyzed with HPLC system. Elution was with 1–20% MeCN in 50 mM ammonium formate buffer for 30 min at a linear gradient with a column temperature 60 °C and a flow rate of 1 mL/min.

## 4. Conclusions

The major factors responsible for the rate of 3-vinyl carbazole based photo-cross-link assisted cytosine deamination at physiological conditions are the hydrophilicity of the photo-cross-linker and the hydrogen bonding of the target cytosine. Thus, the best combination of counter-base and photo-cross-linker can result in the highest conversion of C→U in the least time at physiological conditions, when inosine is used as the counter-base and ^OHV^K is used as the photo-cross-linker.

## Figures and Tables

**Table 1:** The sequence of oligodeoxyribonucleic acid (ODN) containing photo-cross-linker.

Entry	Sequence of ODNs (5′ to 3′)
ODN(G^CNV^K)	AAATGCG^CNV^KACGTCCC
ODN(G^NH2V^K)	AAATGCG^NH2V^KACGTCCC
ODN(G^OHV^K)	AAATGCG^OHV^KACGTCCC
ODN(C^CNV^K)	AAATGCC^CNV^KACGTCCC
ODN(C^NH2V^K)	AAATGCC^NH2V^KACGTCCC
ODN(C^OHV^K)	AAATGCC^OHV^KACGTCCC
ODN(I^CNV^K)	AAATGCI^CNV^KACGTCCC
ODN(I^NH2V^K)	AAATGCI^NH2V^KACGTCCC
ODN(I^OHV^K)	AAATGCI^OHV^KACGTCCC

**Table 2:** Partition coefficient (LogP).

Entry	Retention Time (min)	LogP
ODN(I^OHV^K<>C)	13.8	0.52
ODN(G^OHV^K<>C)	14.2	0.54
ODN(C^OHV^K<>C)	14.7	0.57
ODN(I^NH2V^K<>C)	15.4	0.61
ODN(G^NH2V^K<>C)	16.0	0.64
ODN(I^CNV^K<>C)	16.3	0.66
ODN(G^CNV^K<>C)	16.6	0.68
ODN(C^CNV^K<>C)	17.3	0.72
ODN(C^NH2V^K<>C)	17.8	0.75	

## Supplementary Materials

The MS data, UPLC result and enzymatic digestion of deamination product is available online.
