# Supplementary material for: Study of Photochemical Cytosine to Uracil Transition via Ultrafast Photo-Cross-Linking Using Vinylcarbazole Derivatives in Duplex DNA
Source: Molecules. 2018 Apr 4;23(4):828. doi: 10.3390/molecules23040828 (PMC6017022; doi:10.3390/molecules23040828)
Supplement: Supplementary file 1 [file molecules-23-00828-s001.pdf]

Supplementary information

Study of photochemical cytosine to uracil transition via ultrafast photo-cross-linking using vinylcarbazole derivatives in DNA duplex double

Siddhant Sethi[1], Shigetaka Nakamura[1] and Kenzo Fujimoto[1,*]

1. Department of Advanced Science and technology, Japan Advanced Institute of Science and Technology, 1-1 Asahidai, Nomi, Ishikawa, Japan 923-1211

Contents

1. Mass analysis of ODNs
2. UPLC chromatograms for Log P analysis
3. Log P and deamination rate correlation
4. NMR analysis

1. MALDI-TOF MS analysis of ODNs

Table S1: MALDI-TOF-MS analysis of ODNs containing ^{CNV}K

ODN(XK)	Calculated Mass $(M+H)^+$	Experimental Mass $(M+H)^+$
$C^{CNV}K$	4587.84	4586.43
$C^{NH2V}K$	4605.22	4607.21
$C^{OHV}K$	4592.87	4592.00
$G^{CNV}K$	4627.84	4625.90
$G^{NH2V}K$	4645.33	4644.25
$G^{OHV}K$	4632.08	4630.44
$I^{CNV}K$	4610.97	4608.23
$I^{NH2V}K$	4629.65	4631.02
$I^{OHV}K$	4617.17	4616.19

2. UPLC chromatograms for photo-cross-linking

Photo-cross-linking reaction

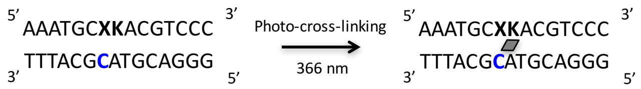

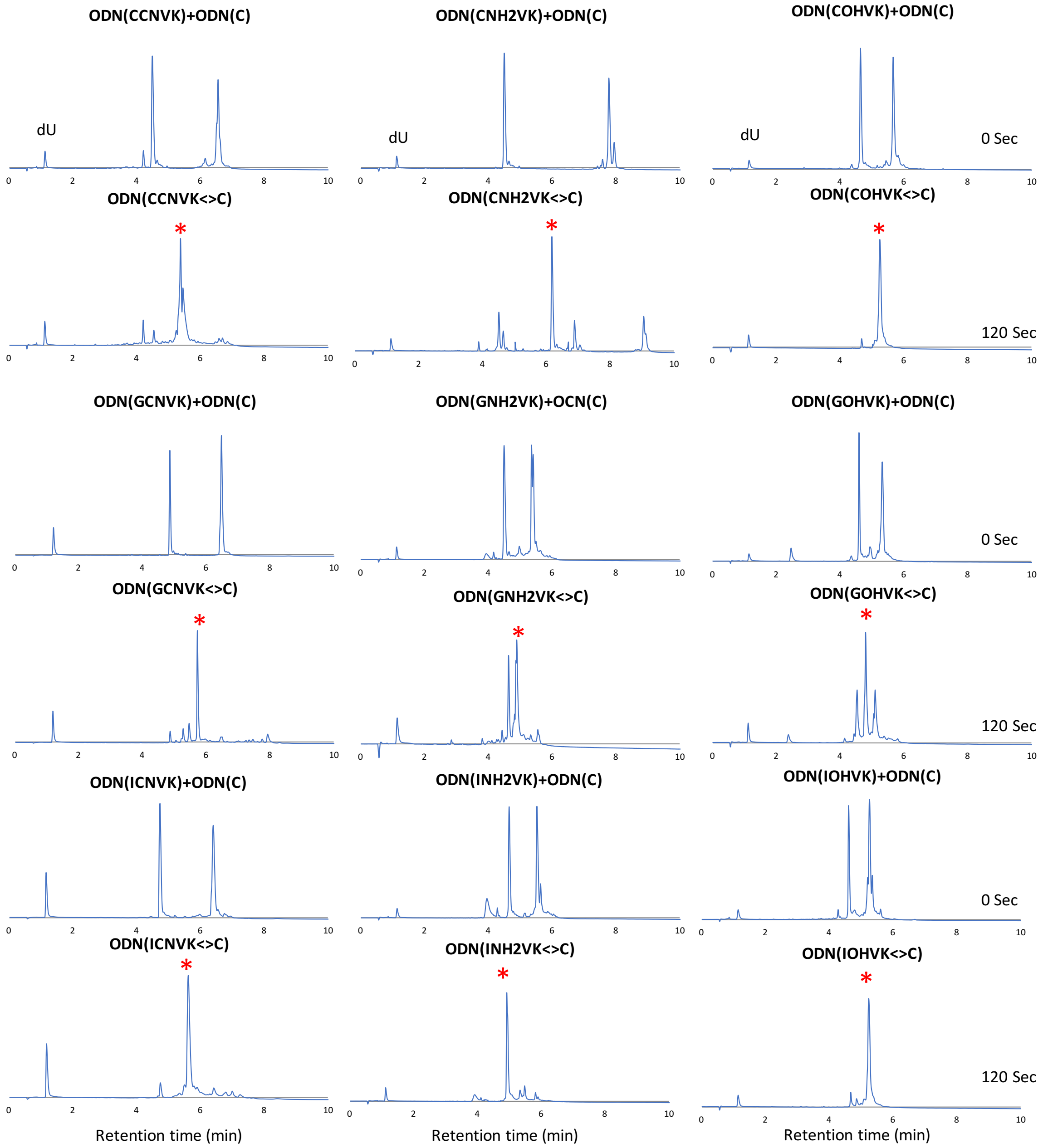

Figure S1**.** UPLC chromatograms of the mixture of OGN(XK) and cODN(C). [ODN] = 10 µM in 50 mM sodium cacodylate buffer (pH 7.4) containing 100 mM NaCl. Photoirradiation at 366 nm was performed at 4ºC. 50 µM 2′-deoxyuridine (dU) was used as an internal standard. Peaks marked with asterisk (*) are the newly formed photo-adducts. X= G, C, I. K= ^{OHV}K and ^{NH2V}K.

3. Enzymatic digestion of ODNs after deamination (3days) and photo-splitting

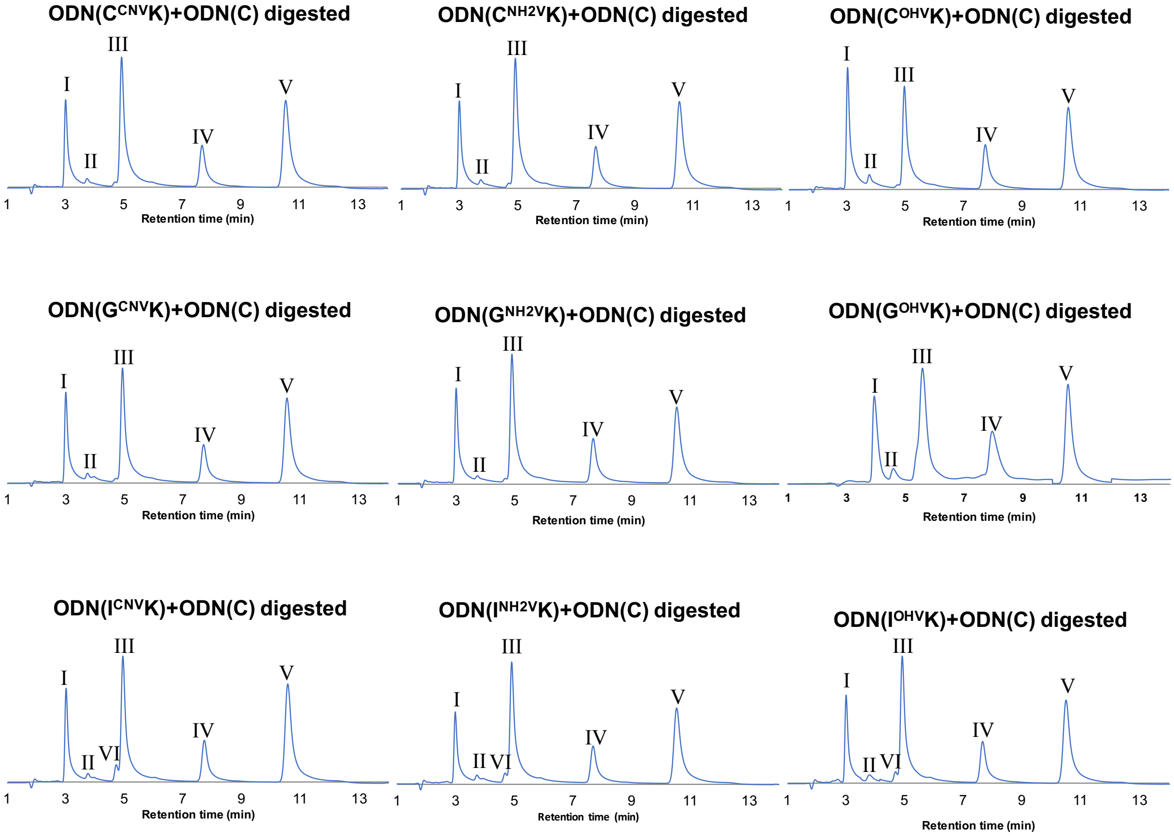

Figure S2: Enzymatic digestion of photo-splitted ODNs after deamination reaction using nuclease P1 and alkaline phosphatase. The peak ratio indicated the amount of nucleoside in the ODN. I=dC, II=dU, III=dG, IV=dT, V=dA, VI=dI. HPLC conditions: 1-20% MeCN in 50 mM ammonium formate, liner gradient, for 30 min, column temperature 60ºC.

4. UPLC chromatograms for Log P calculations

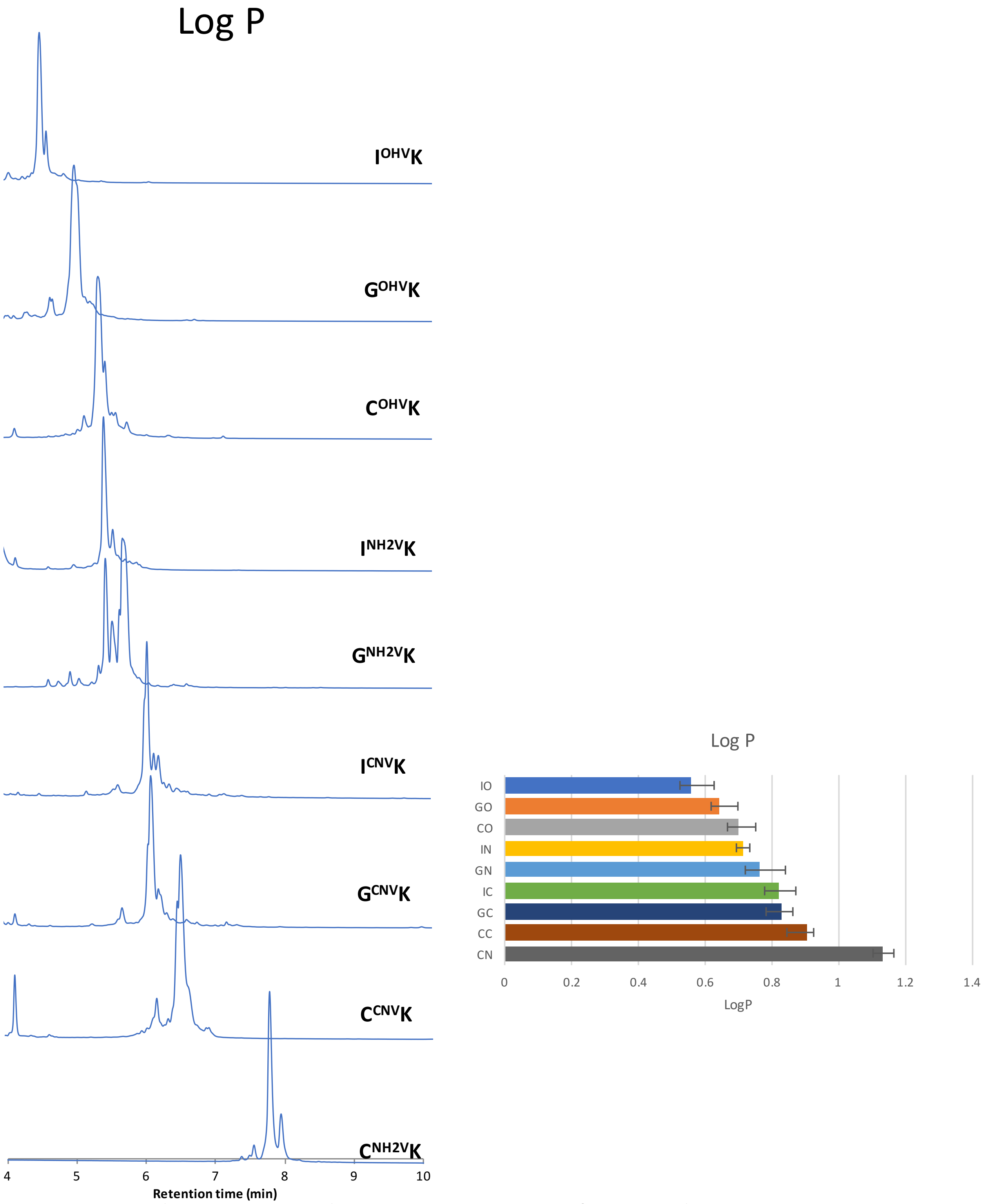

Figure S3: UPLC chromatograms showing retention time of individual ODN in 1 to 15 % MeCN gradient over 12 min linearly in ammonium formate (50 mM). Bar graph showing correlation between ODN and Log P values in increasing order of hydrophobicity.

5. Log P and deamination rate constant correlation

Table S2: Log P and reaction rate constant correlation.

Entry	Retention time (min)	Log P	Deamination rate constant (h^{-1})
$I^{OHV}K$	4.49	0.56	0.0071
$G^{OHV}K$	4.96	0.64	0.0036
$C^{OHV}K$	5.31	0.70	0.0024
$I^{NH2V}K$	5.38	0.71	0.0035
$G^{NH2V}K$	5.66	0.76	0.0025
$I^{CNV}K$	6	0.82	0.0047
$G^{CNV}K$	6.04	0.83	0.0023
$C^{CNV}K$	6.47	0.90	0.0006
$C^{NH2V}K$	7.78	1.13	0.0023

Hydrophilicity (decreasing)

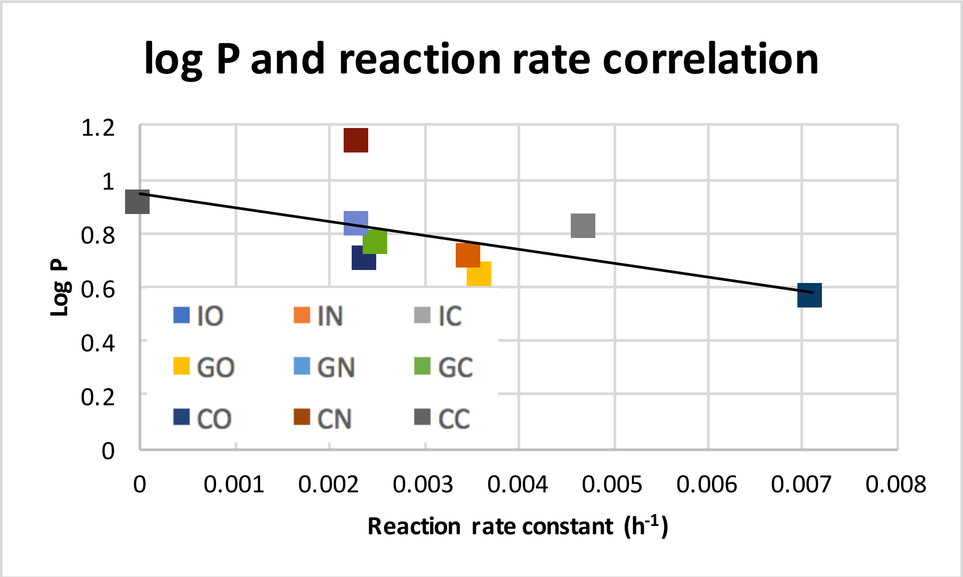

Figure S4: Correlation of LogP and reaction rate constant.
